# PRGminer: harnessing deep learning for the prediction of resistance genes involved in plant defense mechanisms

**DOI:** 10.3389/fpls.2025.1411525

**Published:** 2025-06-03

**Authors:** Naveen Duhan, Rakesh Kaundal

**Affiliations:** ^1^ Bioinformatics Facility, Center for Integrated BioSystems, Utah State University, Logan, UT, United States; ^2^ Department of Plants, Soils, and Climate, College of Agriculture and Applied Science, Utah State University, Logan, UT, United States

**Keywords:** plants, resistance genes, Rgenes, deep learning, CNN, defense mechanism

## Abstract

Plant resistance genes are crucial in plant defense systems against a variety of diseases and pests. These plant-specific genes encode proteins that identify particular molecular patterns associated with pathogens invading the plants. When these resistance genes are active, they initiate a sequence of molecular processes that culminate in the activation of defensive responses such as the synthesis of antimicrobial chemicals, cell wall strengthening, and triggering of programmed cell death in infected cells. Plant resistance genes are exceedingly varied, with several classes and subclasses found across a wide range of plant species. The identification of new resistance genes (Rgenes) is a critical component of disease resistance breeding. Nonetheless, identifying Rgenes in wild species and near relatives of plants is not only challenging but also time-consuming. In this study, we present PRGminer, a deep learning-based high-throughput Rgenes prediction tool. PRGminer is implemented in two phases: Phase I predicts the input protein sequences as Rgenes or non-Rgenes; and Phase II classify the Rgenes predicted in Phase I into eight different classes. Among all the sequence representations tested, the dipeptide composition gave the best prediction performance (accuracy of 98.75% in a *k*-fold training/testing procedure, and 95.72% on an independent testing) with a high Matthews correlation coefficient (0.98 training and 0.91 in independent testing) in Phase I; phase II (overall accuracy of 97.55% in a *k*-fold training/testing and 97.21% in an independent testing) with the MCC values of 0.93 for *k*-fold training procedure and 0.92 in an independent testing. PRGminer is available as a webserver which can be freely accessed at https://kaabil.net/prgminer/, as well as a standalone tool available for download at https://github.com/usubioinfo/PRGminer. PRGminer will help researchers to accelerate the discovery of new R genes, understand the genetic basis of plant resistance, and develop new strategies for breeding plants that are resistant to disease and pests.

## Introduction

Scientists have long been fascinated by plants’ intricate defense mechanisms in the face of ever-evolving plant pathogens and pests. These mechanisms are controlled by a complex network of genes that play an important role in conferring resistance to various pathogens and pests. Novel pathogens and their global spread endangers food security and causes agricultural and economic losses (Ristaino et al., 2021; Skendžić et al., 2021; FAO publications catalogue 2023, 2023). Plant innate immunity is a complex system that protects plants from diseases and pests. Immunity is built on two layers of pathogen recognition: effector-triggered immunity (ETI) and pathogen-associated molecular pattern (PAMP)-triggered immunity (PTI) (Jones and Dangl, 2006; Peng et al., 2018; Ray et al., 2019; Nguyen et al., 2021). PAMPs are recognized by receptors on the cell’s surface in PTI, however infecting organisms may be able to halt PTI signaling by producing effector proteins that limit the activation of defensive responses. The effectors may be identified by the second defense level, which includes resistance (R)-genes that trigger ETI (Andolfo and Ercolano, 2015). R-genes, or plant resistant genes encode proteins that detect particular pathogen effectors and elicit a quick and powerful immune response. Understanding the processes and roles of these genes is not only critical for unraveling the complex biology of plants, but it also holds enormous potential for devising long-term strategies to safeguard crops and assure food security in the face of rising agricultural challenges. Therefore, the correct identification and classification of R-genes in plant genomes is critical (Capistrano-Gossmann et al., 2017; Andolfo et al., 2021b, a).

Plant R-genes are classified into two types: membrane-bound pattern recognition receptors (PRRs) and intracellular resistance receptors. PRRs are made up of two types of receptors: receptor-like proteins (RLPs) and receptor-like kinases (RLKs), and they are found on the plant plasma membrane as the first layer of the surveillance system to detect microbe-derived molecular patterns. PRRs are known to have extremely varied extracellular domains such as the lysin motif (LYK), the leucine-rich repeat (LRR), and the lectin receptor like kinase (LECRK) (Zhou and Yang, 2016). The vast majority of intracellular resistance receptors (NBS-LRRs or NLRs) are nucleotide-binding sites (NBSs) and LRR proteins that can identify pathogen-delivered effectors. These proteins are divided into two subclasses based on the N-terminal domain: CC-NBS-LRR (CNL) contains a coiled-coil domain at N-terminal region with a NBS domain which is part of the NB-ARC domain and LRR domain, and the TIR-NBS-LRR (TNL) which contains an interleukin-1 Receptor (IL-1R) at the N-terminal region in place of coiled-coil domain. Plants R-gene defensive arsenal is made up of both incomplete (single or fragmented domains) and full-length (NB-LRR) genes. Although incomplete genes may be called pseudogenes, they are frequently expressed and may play a role in the regulation of full-length R-genes (Van Ooijen et al., 2008; Andolfo et al., 2019, 2020; Han, 2019; Sun et al., 2020b).

Plant resistance genes are often organized in clusters of closely duplicated genes, though they may also exist as individual units scattered across the genome (Di Donato et al., 2017; Barchi et al., 2019). The current automatic gene annotation methods face challenges in accurately predicting and identifying R-genes loci due to their unique genomic structure within the gene clusters. This often leads to incomplete and fragmented annotations (Jupe et al., 2013; Andolfo et al., 2014). The presence of numerous similar sequences can hinder local genome assembly process and can cause issues with gene annotation (Tørresen et al., 2019). The situation is further complicated by the fact that R-genes are typically expressed at low levels, making it difficult to predict genes using only the RNA sequencing (RNA-Seq) data. Furthermore, because R-genes can be mistaken for repetitive sequences, using public databases for transposable elements (TEs) in the genome annotation processes may obscure the detection of R-genes loci. As a result, finding R-genes in the previously uncharacterized organisms is challenging (Marone et al., 2013).

Several tools have been reported for the prediction of plant R-genes. Most of the tools are alignment-based (Steuernagel et al., 2015; Li et al., 2016; Restrepo-Montoya et al., 2020; Andolfo et al., 2022). Some of them are based on motif search and alignment, others uses program such as BLAST (Altschul et al., 1990; Camacho et al., 2009), InterProScan (Zdobnov and Apweiler, 2001), HMMER3 (Eddy, 2011), nCoil (Lupas et al., 1991), Phobius (Käll et al., 2004), SignalP 4.0 (Petersen et al., 2011), TMHMM2 (Krogh et al., 2001), and PfamScan (Finn et al., 2014) to predict domains in the protein sequences and assign them to R-genes classes. However, similarity-based methods fail in case of low homology. This might be particularly true when annotating the newly sequenced plant genomes. Some other methods use the traditional machine learning approaches for domain prediction. These methods extract various numerical features from the protein sequences and are fed into the machine learning framework such as the support vector machines (SVM) for R-genes prediction (Kushwaha et al., 2016, 2021; Pal et al., 2016; Wang et al., 2021).

In this study, we present PRGminer, a cutting-edge deep learning-based tool designed specifically for the accurate prediction of resistance proteins. Deep Learning is a class of machine learning algorithms that uses multiple layers to extract higher-level features from the raw input data. The derived protein sequence is used as an input, extracting sequential and convolutional features from raw encoded protein sequences based on classification rather than traditional alignment-based methods for R-genes prediction. PRGminer provides a comprehensive approach to identifying and classifying R-genes that outperforms previous methods in terms of efficacy and precision. PRGminer has been rigorously tested and validated, and it performs exceptionally well in predicting experimentally validated R-genes. By harnessing the power of deep learning, this innovative tool opens new possibilities for exploring and understanding the genomic landscape of resistance mechanisms in various organisms. Its successful identification of known R-genes is an important step toward realizing the full potential of this cutting-edge technology in furthering our understanding of immunity and defense systems.

## Materials and methods

**Figure 1:**
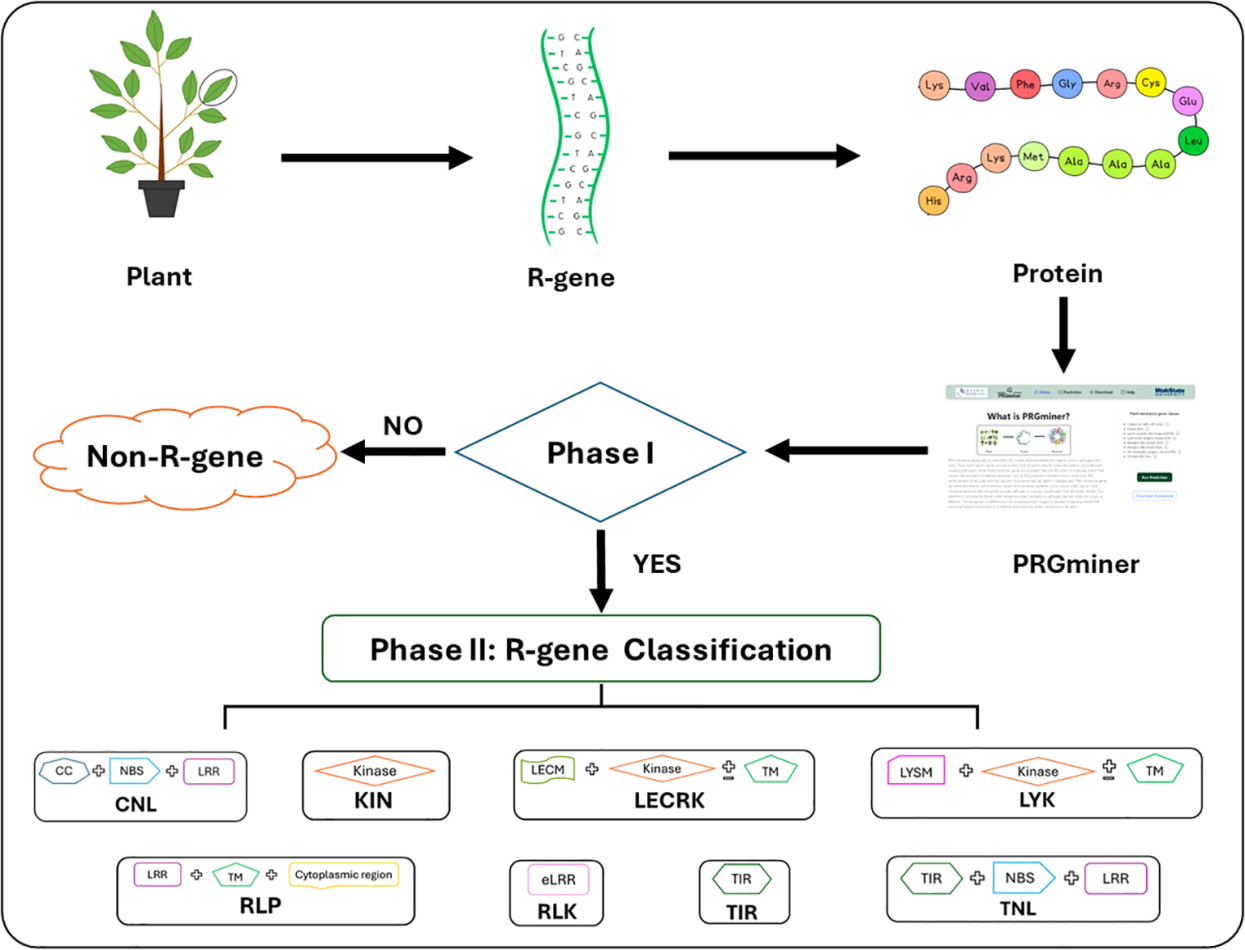
The overall workflow of PRGminer for identifying and classifying R-genes in plants. The tool performs in two phases: In Phase I, an input protein sequence is classified as Plant R-gene vs non-R-genes. If a protein is identified as non-R-gene it is excluded from further analysis. However, if the protein is classified as an R-gene, it proceeds to Phase II where it undergoes further classification. In Phase II, R-genes are categorized based on specific domain structures, helping to distinguish various subtypes. These subtypes include: CNL (Coiled-coil, Nucleotide-binding site, Leucine-rich repeat domains), KIN (Kinase domain), RLP (Leucine-rich repeat and Transmembrane domains with a cytoplasmic region), LECRK (Lectin, Kinase, and Transmembrane domains), RLK (Extracellular Leucine-rich repeat and Kinase domains), LYK (LysM domain, Kinase, and Transmembrane domains), TIR (Toll/interleukin-1 receptor domain), and TNL (Toll/interleukin-1 receptor, Nucleotide-binding site, and Leucine-rich repeat domains).

PRGminer is implemented in two phases: Phase I identifies a query protein as resistance gene or non- resistance gene; Phase II further classifies the predicted resistance gene into one of the eight R-gene classes. The overall workflow for the development of PRGminer is depicted in **Figure 1**.

### PRGminer training datasets

**Figure 2:**
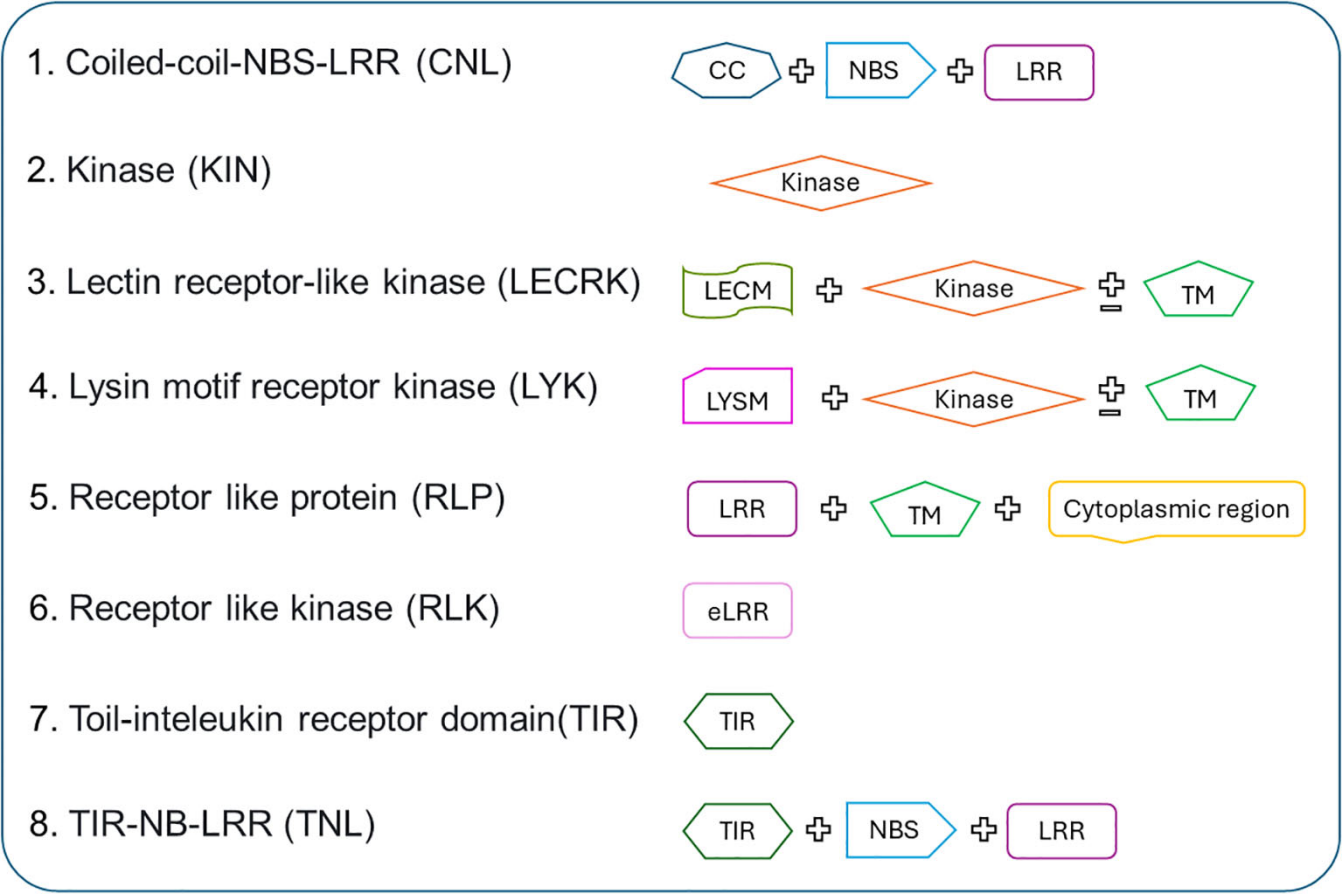
The architecture of R-genes classified in PRGminer. Where R-genes are classified into different families based on domains present in them: CNL (Coiled-coil, Nucleotide-binding site, Leucine-rich repeat domains), KIN (Kinase domain), RLP (Leucine-rich repeat and Transmembrane domains with a cytoplasmic region), LECRK (Lectin, Kinase, and Transmembrane domains), RLK (Extracellular Leucine-rich repeat and Kinase domains), LYK (LysM domain, Kinase, and Transmembrane domains), TIR (Toll/interleukin-1 receptor domain), and TNL (Toll/interleukin-1 receptor, Nucleotide-binding site, and Leucine-rich repeat domains).

The R-genes and non-Rgenes protein sequence datasets were downloaded from various public databases such as Phytozome (Goodstein et al., 2012), Ensemble plants (Yates et al., 2022) and NCBI (https://www.ncbi.nlm.nih.gov/). CD-HIT (Li and Godzik, 2006; Fu et al., 2012) was used on these datasets to eradicate the redundant sequences. Sequences were then filtered based on the domain (NB-ARC, TIR, CC, kinase, LRR, Serine/threonine-LRR, and Kinase-LRR) information available from Ensemble BioMart (Kinsella et al., 2011) and Phytozome Biomart (Goodstein et al., 2012) and the dataset generated from this is designated as R-genes (positive dataset)and all other sequence which does not contain any domain were designated as non-Rgenes (negative dataset). For phase I, the dataset (18,952 R-genes and 19,212 Non-Rgenes) was divided into training and independent testing in the ratio of 9:1; means 90% of the data from both the R-genes and non-Rgenes was used for the *k*-fold training/testing procedure, and the remaining 10% was kept separate to be used as an independent dataset for benchmarking of the models. In phase II, the R-genes dataset was divided into eight classes: Coiled-coil-NBS-LRR (CNL) (1883 sequences), Kinase (KIN) (8591 sequences), Lysin motif receptor kinase (LYK) (902 sequences), Lectin-receptor like kinase (LECRK) 117 sequences, Receptor like protein (RLP) (1802 sequences), Receptor-like kinase (RLK) (4362 sequences), Toil-interleukin receptor domain (TIR) (511 sequences), and interleukin-1 Receptor (IL-1R)- NBS-LRR (TNL) (784 sequences). These categories were selected based on their biological relevance and sufficient sequence representation, ensuring the development of a robust predictive model. The dataset was then split following the 9:1 ratio training and independent datasets. The overall architecture of 8 R-gene classes is presented in **Figure 2**. Although PRGdb classifies R-genes into more categories based on fragmented domain sequences, which lack sufficient sequence representation for training data require for training a robust model. So we included only the classes having a good number for training data to maintain the model accuracy and stability. For additional validation, a second independent dataset was prepared by downloading more experimentally validated plant resistance proteins from the PRGdb database (Calle García et al., 2022). In this way, we created two independent datasets for efficient benchmarking of the deep learning-based prediction models. The total number of sequences downloaded and used for training are presented in **Table 1**.

**Table 1:** Number of sequences used for training and testing in phase I and phase II.

Phase I Data		
Class	Sequences	
	CD-hit 100%	CD-hit 40%
Rgenes	79715	18952
Non-Rgenes	81230	19212

Phase II Data		
R-gene Class	Sequences	
	CD-hit 100%	CD-hit 40%
CNL	5898	1883
KIN	37198	8591
LYK	5424	902
LECRK	702	117
RLP	12820	1802
RLK	15957	4362
TIR	1345	511
TNL	371	784

### PRGminer sequence representation

Machine learning algorithms often demand fixed-size input data. Deep learning algorithms, on the other hand, provide a solution by eliminating the requirement for consistent manual dimensioning and handmade characteristics. This is especially useful when data volumes and complexity grow. The deep learning system does feature reconstruction and classifier training effectively at the same time. We explored several sequence representations for the model training in all phases of our work. The top two best sequence representations are discussed below, while the training statistics for all other alternate representations are presented in **Supplementary File 1**.

For both phase I and phase II, the two best sequence representations were dipeptide composition (DPC) and a hybrid of DPC and Normalized Moreau-Broto (NMBroto). The DPC (Dipeptide Composition) approach has been identified as a way for thoroughly representing global information for each protein sequence while making use of the sequence order effects. This fixed-pattern value, which generally consists of 400 (20 x 20), efficiently reflects the protein structural properties and local amino acid order. The fraction of each dipeptide was calculated using the equation below.


$$f\left(r,s\right)=\frac{N_{rs}}{N-1}r,s=1,2,\ldots ,20$$

where *N_rs_* is the number of dipeptides represented by the amino acid type *r* and type s , and N is the length of the sequence. The other sequence representation, Normalized Moreau–Broto is a feature derived from autocorrelation, which quantifies the association between two objects (protein or peptide sequences) by considering their specific structural or physicochemical properties distributed along the amino acid sequence. It measures how these properties are correlated at varying intervals within the sequence. The NMBroto autocorrelation precisely characterizes these associations and provides valuable insights into the relationships between amino acid properties and the overall structure of the sequences under study. The NMBroto generates a vector of length 240 calculated using the equation below:


$$I\left(d\right)\;=\;\frac{\sum_{i=1}^{N-d}\left(P_{i}*P_{i+d}\right)}{N-d}\;d=1,2,\ldots ,30$$

where *d* is the lag of the autocorrelation, *P_i_* is the value of *i_th_* amino acid in a property entry of AAindex.

### Model training architecture

**Figure 3:**
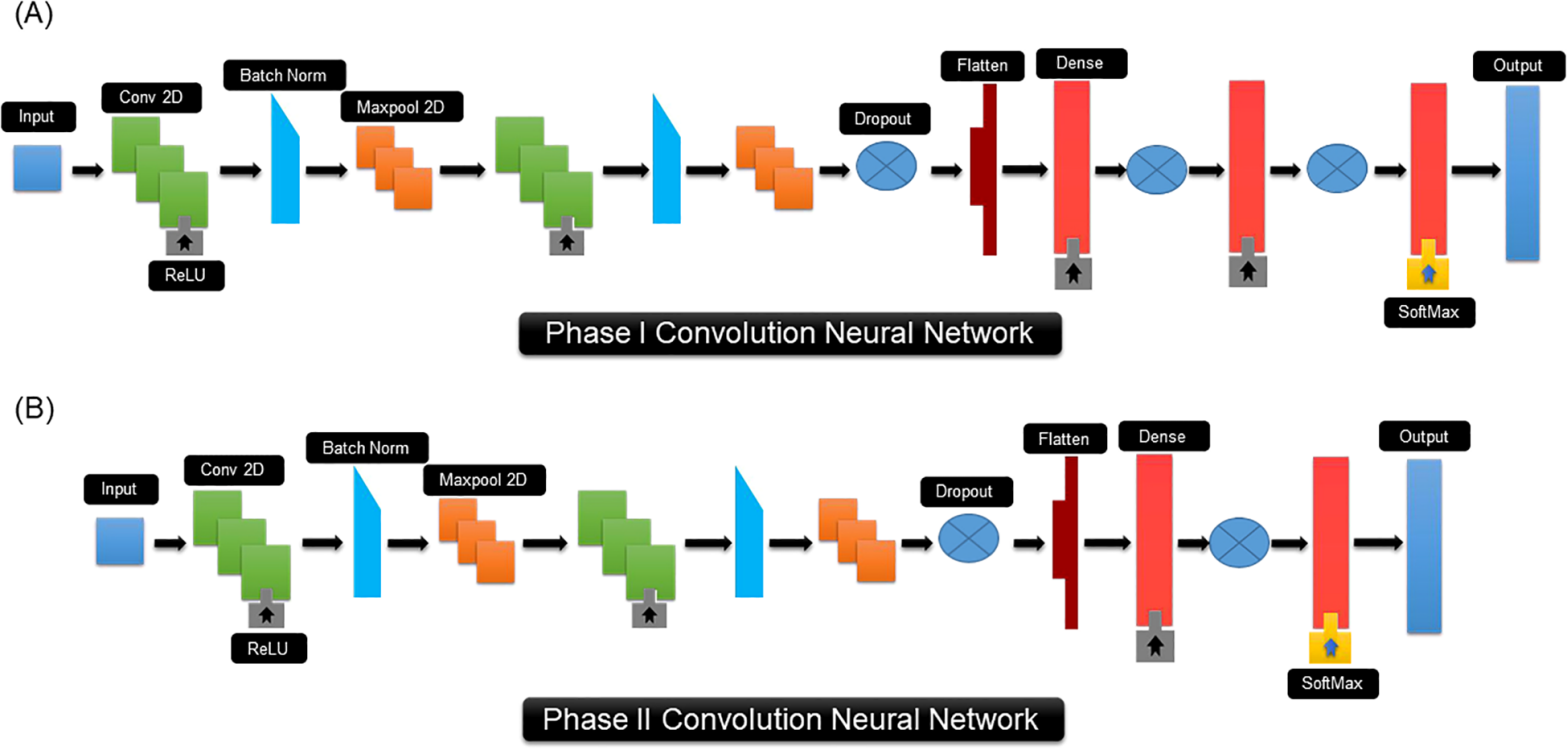
CNN architecture of PRGminer. **(A)** CNN architecture used for Phase I Rgene vs Non-Rgene. **(B)** CNN architecture used of Phase II Rgene classification. The different terminology used in the figure are explained here: Input: This represents the input sequence vector for training; Conv 2D is a 2-dimensional convolution kernel that, when convolved with the layer input, yields a tensor of outputs; Batch Norm: Batch normalization is a training strategy for very deep neural networks that standardizes the inputs to each mini-batch; ReLU: Rectified linear unit; Maxpool2D: Max pooling operation for 2D spatial data; Dropout: Dropout refers to the practice of randomly ‘dropping out,’ or omitting, units during the neural network training process; Flatten: a flattened layer to process data into one-dimensional array. Dense: a deep neural network layer which each neuron receives input from all the neurons; Softmax: Softmax is a mathematical function that transforms a number vector into a probability vector; Output: output prediction probabilities.

PRGminer contains two separate convolution neural networks (CNNs) that conduct two distinct classification tasks with protein sequences as input data. The CNN for phase I consists of two 2D convolution layers, two max-pooling layers, three dropout layers, two batch normalization, one flattening layer and three fully connected layers that are finally fed to an output, see **Figure 3** for details. The initial layer in a CNN is always a convolutional layer. For our PRGminer-specific CNN, the first layer involves a sequence representation length vector upon which 2D convolutional operations are applied, using default parameters like n × n kernel size, *f* filters, 1 × 1 steps, and 1 × 1 zero-padding. This convolutional operation filters the essential features of the motif. To optimize the network, we experimented with various hyperparameters and identified suitable choices. Additionally, to enhance the efficiency and prevent overfitting, we introduced a 2D max-pooling layer with a 1 × 1 stride to reduce the matrix calculation size and remove non-maximal values. Before proceeding to the three fully connected layers, we incorporated a flattening layer to transform the input into a suitable format. The first fully connected layer comprises of 512 hidden nodes, followed by a second layer with 256 hidden nodes, and finally, a third fully connected layer was introduced with 2 hidden nodes, facilitating the binary classification for the PRGminer phase I model. This well-structured CNN architecture ensures accurate and efficient classification of the PRGminer phase I models (**Figure 3**). To achieve a successful classification in phase-II, the CNN design for phase II contains many layers, **Figure 3**. It consists of three 2D convolution layers, two max-pooling layers, two dropout layers, one batch normalization, one flattening layer, and three dense layers, with an output layer at the end. Our CNN first layer is composed of 640-size vector on which we perform diverse 2D convolutional operations with default settings such as the n x n kernel size, *f* filters, 1 x 1 steps, and 1 x 1 zero-padding. In addition, for better efficiency, we used a 2D max-pooling layer with a 1 x 1 stride. We also included a flattening layer before the three fully linked layers to flatten the input. The PRGminer phase II models are binary classified using a first fully connected layer with 512 hidden nodes, followed by a second fully connected layer with 8 hidden nodes using the softmax activation (**Figure 3**).

In the development of PRGminer, we used a uniform distribution to randomly initialize the neural network weights. PRGminer used ReLU (Rectified Linear Unit) as the activation function for both the convolutional layers and the hidden layers of the fully connected layers in each CNN. The SoftMax function was employed as the activation function for the output layer of the completely linked layers.


f(x) = max(0,x)

$$\text{σ}{\left(Z\right)}_{i}=\frac{e^{Z_{i}}}{\sum_{j=1}^{K}e^{Z_{i}}}$$

Batch normalization was used to address any internal-covariate shift and to stabilize the learning process. This normalization strategy ensures that each layer’s input distribution approximates a typical Gaussian distribution, resulting in improved convergence and reducing overfitting in the deep learning process. Each neuron learns the feature representation of the input signal across the period depending on their learning abilities, resulting in varying learning rates for each neuron of the network to maximize the objective function. Therefore, the SGD optimizer was used as the optimizer feature in this work. The categorical entropy of loss function was used to train the neural network. The CNN architecture in the phase-I and phase-II were trained up to 100 epochs.

### Performance evaluation

To evaluate the predictive performance of the phase I and phase II classification models using the sequence representations, we employed a 10-fold cross-validation procedure to develop our models and evaluated the training performance with certain statistical parameters. Further, two independent datasets were used to assess the capability of our models. In a typical supervised binary classification problem, the evaluation process involves mapping each query point from the test set to its correct class label. The classifier then assigns these points to one of the following categories: true positive (TP), true negative (TN), false positive (FP), or false negative (FN). For multiclass classification, a one-on-one approach is utilized to determine these categories for each class. Here, each query point is considered as a positive or negative point for a specific class. Using this approach, TP, TN, FP, and FN are calculated for each class. Several performance metrics were used, including Specificity, Sensitivity, Precision, Accuracy, F1-score, and Matthews correlation coefficient (MCC) (Fang et al., 2018; Duhan et al., 2022; Kaundal et al., 2022). The motivation for calculating MCC (Matthews correlation coefficient) is to address situations where accuracy and specificity may overstate the classifier’s performance. MCC provides a more robust measure by taking into account both the true positive and true negative rates. A MCC value of +1 indicates an optimal prediction, 0 denotes random prediction, and -1 signifies a complete disagreement between the correct and predicted classes. Thus, MCC serves as a reliable indicator of classifier performance, offering a balanced assessment of its predictive capabilities. These evaluation metrics are defined as below:


$$Sensitivity\;=\;\frac{\left(TP\right)}{\left(TP+FN\right)}$$

$$Specificity\;=\;\frac{\left(TN\right)}{\left(TN+FP\right)}$$

$$Precision\;=\;\frac{\left(TP\right)}{\left(TP+FP\right)}$$

$$Accuracy\;=\;\frac{\left(TP+TN\right)}{\left(TP+TN+FP+FN\right)}$$

$$F1-score\;=\;2*\frac{\left(Precision*Sensitivity\right)}{\left(Precision+Sensitivity\right)}$$

$$MCC\;=\;\frac{\left(TP*TN\right)-\left(FP*FN\right)}{\sqrt{\left(TP+FN\right)*\left(TP+FP\right)*\left(TN+FP\right)*\left(TN+FN\right)}}$$

### Development environment and webserver development

For training the deep learning models, we employed the CNN architecture using Keras (version 2.4.0) Python package with TensorFlow (version 2.2.0) as backend (Abadi et al., 2016). Python module *scikit- learn* was used to create a confusion matrix (Pedregosa et al., 2011).

The PRGminer webserver was developed using PHP 7.4, Javascript, JQuery and html and is hosted on a high-performance computing cluster. These technologies offer an intuitive and responsive platform for users, ensuring seamless communication between the frontend and backend. This technology stack provides us a robust foundation, enabling the web server to efficiently handle multiple user requests in parallel, process the data, and deliver accurate results.

## Results and discussion

Among several deep learning approaches considered during the development of PRGminer, CNN was chosen for its demonstrated effectiveness in identifying the functional regions such as motifs and domains, as well as classifying enzymes/non-enzymes in biological sequences, particularly protein sequences (Duhan et al., 2022; Kaundal et al., 2022). CNN was chosen because of its proven track record in processing biological data and extracting significant features, making it a dependable and strong tool for sequence analysis and classification tasks.

The phase I of PRGminer first identifies the input protein sequences into R-genes or non-R-genes. The performance evaluation metrics of phase I are depicted in **Table 2**. The phase I classifier performance was demonstrated by the average 10-fold training/testing: specificity (98.86%), sensitivity (98.65%), precision (99.02%), Accuracy (98.75%), F1- score (0.99) and MCC (0.98). On an independent dataset-I (testing of the models on data not used in training; 10% data as kept separate initially), we again got a high specificity (96.35%), sensitivity (95.17%), precision (96.82%), accuracy (95.72%), F1- score (0.96) and a high MCC value (0.91). The phase II model’s classification performance for the correct classification of the identified R-genes into one of the eight classes was demonstrated by the overall average 10-fold training with a high specificity (98.79%), sensitivity (94.70%), precision (95.16%), accuracy (97.55%), F1-score (0.95) and MCC (0.93) values; and on the 8,309 independent sequences, the performance is; specificity (98.55%), sensitivity (94.03%), precision (94.52%), accuracy (97.21%), F1- score (0.94) and a very good MCC value (0.92). The phase II training/testing results are presented in **Table 3**, and results for the independent datasets are presented in **Table 4**. In our evaluation of the PRGminer phases, we recognized that accuracy alone is not sufficient to measure the classification performance of the models, as the accuracy paradox can distort its reliability. Therefore, we also calculated the F1-score for each corresponding phase (Abma, 2009; Valverde-Albacete et al., 2013). The F1-score considers both precision and recall, providing a harmonic description of the classifier’s ability to detect true positive samples, making it a more appropriate measure than accuracy alone. However, it is essential to note that the F1-score might overestimate performance when dealing with unbalanced test data (Boughorbel et al., 2017). Looking at the F1-scores for each PRGminer phase (I and II), as presented in **Tables 2** to **4**, we observed consistently high performance across all classes. Additionally, we assessed the models using a balanced success metric, the MCC score, which was found to be consistently above *≥* 0.90 for all the classes in each phase. This MCC score demonstrates the higher predictive efficiency of our PRGminer models, ensuring their reliability and accuracy in classifying and predicting the plant resistance genes.

**Table 2:** Phase I 10-Fold training and independent testing metrics.

Metrics	Training average 10-FOLD	Independent testing
Sensitivity (%)	98.65	95.17
Specificity (%)	98.86	96.35
Precision (%)	99.02	96.82
Accuracy (%)	98.75	95.72
F1-score (%)	0.99	0.96
MCC	0.97	0.91

**Table 3:** Phase II 10-Fold training/testing metrics.

Metrics	Sensitivity (%)	Specificity (%)	Precision (%)	Accuracy (%)	F1-score (%)	MCC
CNL	96.44	99.70	96.07	99.46	0.96	0.96
KIN	93.47	98.46	97.97	96.25	0.96	0.93
LYK	100.00	99.92	90.48	99.92	0.95	0.95
LECRK	96.67	97.69	75.38	97.62	0.85	0.84
RLP	95.13	99.35	96.45	98.69	0.96	0.95
RLK	95.15	98.80	95.09	98.09	0.95	0.94
TIR	91.67	99.74	85.27	99.61	0.88	0.88
TNL	98.10	99.83	96.26	99.76	0.97	0.97
**Overall**	**94.70**	**98.79**	**95.16**	**97.55**	**0.95**	**0.93**

Bold values display the overall metrics across different classes.

**Table 4:** Phase II independent testing metrics.

Metrics	Sensitivity (%)	Specificity (%)	Precision (%)	Accuracy (%)	F1-score (%)	MCC
CNL	96.95	99.62	95.17	99.43	0.96	0.96
KIN	92.69	98.04	97.46	95.64	0.95	0.91
LYK	90.14	99.93	91.43	99.84	0.91	0.91
LECRK	95.95	97.57	73.38	97.46	0.83	0.83
RLP	95.16	99.26	95.91	98.63	0.96	0.95
RLK	94.67	98.66	94.38	97.89	0.95	0.93
TIR	82.22	99.67	80.43	99.39	0.81	0.81
TNL	98.39	99.84	96.57	99.77	0.97	0.97
**Overall**	**94.03**	**98.55**	**94.52**	**97.21**	**0.94**	**0.92**

Bold values display the overall metrics across different classes.

**Figure 4:**
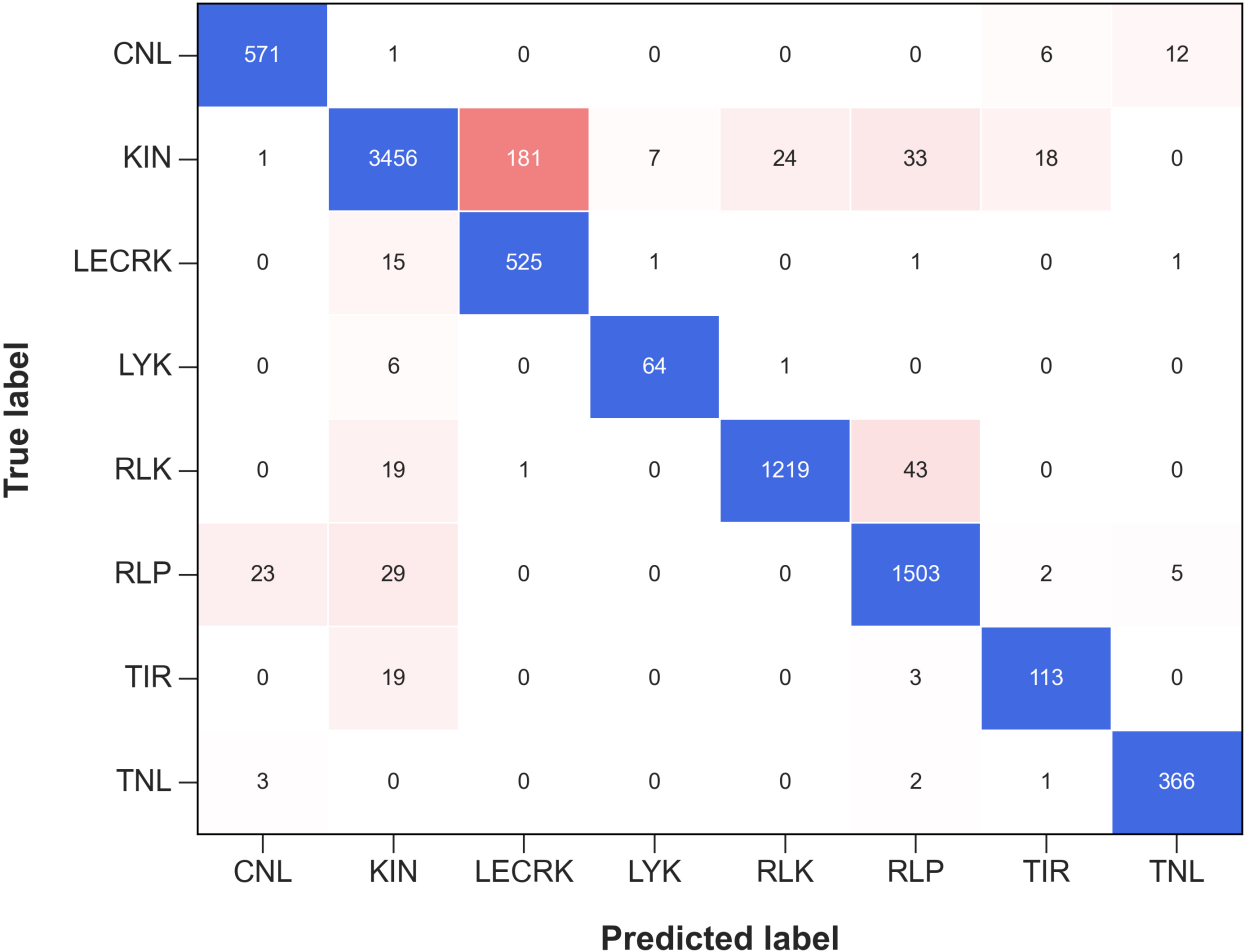
The confusion matrix represents the performance of Phase II Independent Test in classifying various subfamilies of R-genes. The matrix shows how the predicted labels for the different subfamilies (on the x-axis) compare with the true labels (on the y-axis). The blue color represents the correctly predicted true labels while the tomato color gradient represents the number of sequences incorrectly classified into other subfamilies.

Further, we plotted the Confusion Matrix for phase II to examine the performance and efficacy of each R-genes class (Stehman, 1997). A confusion metrics of phase II independent test data is depicted in **Figure 4**. Confusion matrices provide valuable information about the performance of a classification model, beyond just the overall accuracy. They can reveal the hierarchy of prediction classes, showing not only the primary prediction but also the next best prediction and subsequent rankings. This hierarchical view is particularly useful for multi-class classification problems, where a given instance may belong to one of several possible categories. From the **Figure 4**, it can be deduced that almost all of the 8 sub-classes of R-genes are correctly predicted. For example, out of the 3720 sequences for the KIN R-genes in the independent dataset, 3456 are correctly predicted, an accuracy of 92.90%. The remaining 264 sequences (3720 – 3456 = 264) are predicted as 181 (4.87%) into LECRK subclass of R-genes, followed by 33 sequences (0.89%) into the RLP subclass, and 18 sequence (0.04%) in TIR subclass. It has been reported that both KIN R-genes and LECRKs are involved in plant defense against pathogens, and they both encode proteins that contain a kinase domain. Kinase domains are responsible for phosphorylating other proteins, which can activate or deactivate them (Wang and Bouwmeester, 2017; Sun et al., 2020a).

Similarly, out of total 1282 sequences for RLK R-genes class in the independent dataset, 1219 are correctly predicted, an accuracy of 95.09%. The remaining 63 sequences out of 1282 sequences are predicted 43 (3.35%) into RLP class of R-genes, followed by 19 sequence (1.48%) into KIN class. For a total of 1596 RLP sequences in independent dataset, 1503 are correctly predicted with an accuracy of 94.17%. The other 96 sequences were classified into RLK 34 (2.13%), KIN 29 (1.81%), CNL 23 (1.44%), TNL 5 (0.31%), and TIR 2 (0.12%) respectively. Plant receptor proteins play crucial roles in plant immunity, growth, and development. These proteins, which include receptor-like kinases (RLKs) and receptor-like proteins (RLPs), act as pattern recognition receptors (PRRs) to detect microbe- and host-derived molecular patterns, which triggers the first layer of plant defense (Tang et al., 2017).

### Receiver operating characteristics curves analysis

**Figure 5:**
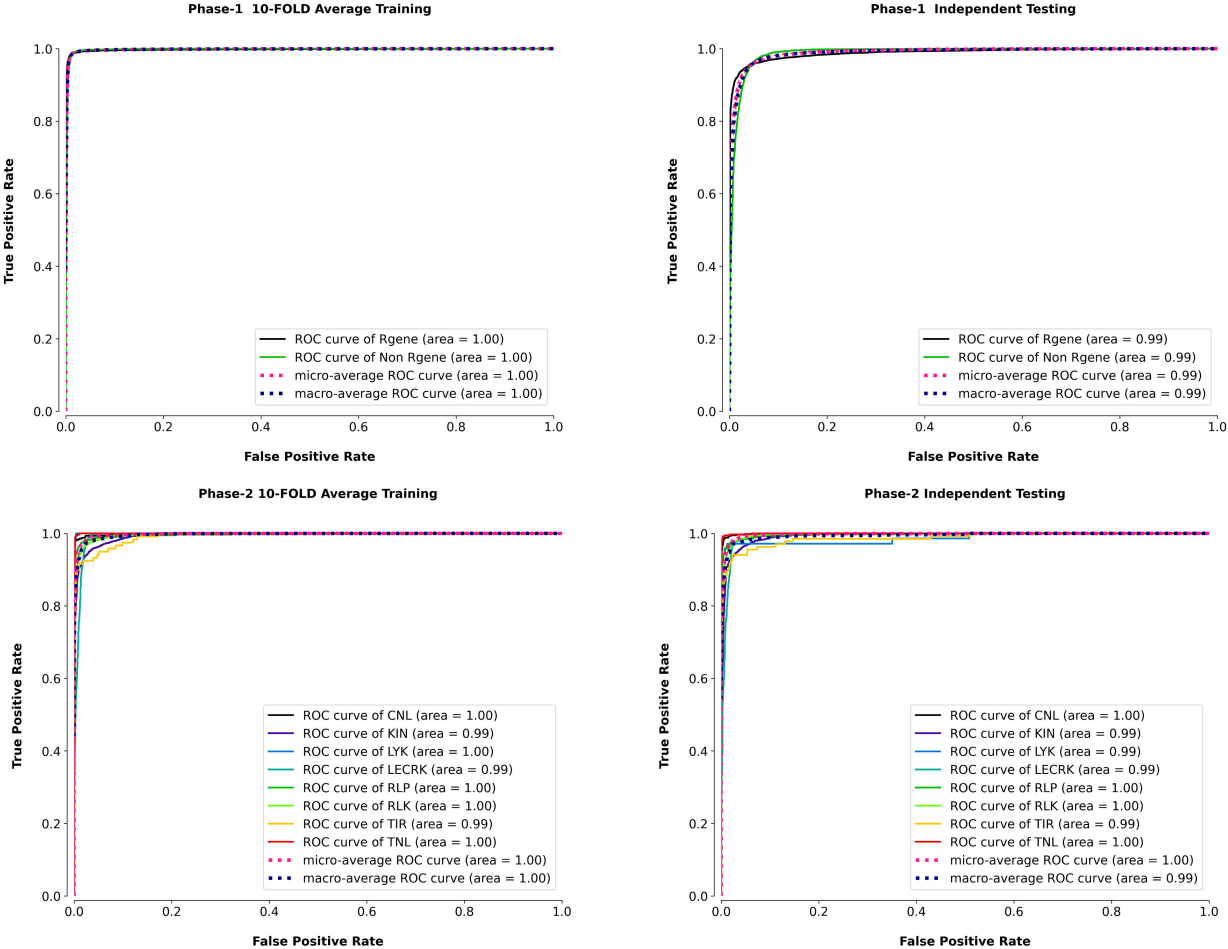
Receiver Operating Characteristic (ROC) curves to evaluate the true positive rate (sensitivity) against the false positive rate, for both Phase 1 (R-gene identification) and Phase 2 (R-gene classification), using 10-fold cross-validation and independent testing. Top Row: ROC curves for Phase 1, which aims to distinguish R-genes from non-R-genes. The left panel shows the average 10-fold cross-validation performance, while the right panel depicts the results on independent testing data. The Area Under the Curve (AUC) values for both datasets are close to 1.0, indicating excellent performance in identifying R-genes (black line) and non-R-genes (green line). Bottom Row: ROC curves for Phase 2, where the goal is to classify R-genes into specific subtypes. The left panel represents the average 10-fold cross-validation performance across different R-gene classes, and the right panel shows the results on independent testing data. Each curve corresponds to a different R-gene subtype: CNL (black), KIN (blue), LYK (green), LECRK (yellow), RLP (teal), RLK (purple), TIR (red), and TNL (orange). The AUC values for all classes are very high, indicating strong performance for each subtype classification.


The micro-average ROC of the model was also plotted along with eight classes in phase II to demonstrate PRGminer models’ average ROC efficiency. The ROC curves of the k-fold training/testing and independent data for phase I and phase II are depicted in **Figure 5**. The PRGminer models’ micro average ROC metric was plotted against the micro average FPR (FPRµ) and micro average TPR (TPRµ), where FPRµ and TPRµ reflected the contribution of all eight classes.

$$TPR_{\text{μ}}\;=\;\frac{\sum_{i=1}^{k}TP_{i}}{\sum_{i=1}^{k}TP_{i}+FN_{i}}$$

$$FPR\text{μ }=\;\frac{\sum_{i=1}^{k}FN_{i}}{\sum_{i=1}^{k}TP_{i}+FN_{i}}$$

The ROC curve is a graphical statistic used to assess a classification model’s performance. It depicts the relationship between the true positive rate (TPR) and the false positive rate (FPR or 1-specificity) at different decision thresholds. The FPR is often plotted against the x-axis, whereas the TPR is shown against the y-axis. The classification model’s FPR is a measure of false-positive predictions based on all negative examples. The TPR, on the other hand, indicates the true positive predictions among all positive cases. The best classification condition of the model is shown by the top-left corner of the ROC curve, when sensitivity and accuracy both approach 100%. The diagonal line from (0, 0) to (0, 1), on the other hand, depicts the classification model’s random output. To have a successful classification pattern, the model’s ROC curve should be above the diagonal line. In other words, the area beneath the curve indicates the performance of the model, and the greater this area, the better the classifier performance. In our results the AUC was 1.0 for 10-fold training in phase I and 0.99 for the independent dataset, indicating that the model performed very well in both cases. Similarly, in phase II, the AUC was 1.0 for 5 classes (CNL, LYK, RLP, RLK, and TNL) and 0.99 for 3 classes (KIN, LECRK, TIR), indicating that the model also performed very well on these datasets. In the independent dataset, the AUC was 1.0 for 4 classes (CNL, RLP, RLK, TNL) and 0.99 for 4 classes (KIN, LYK, LECRK, TIR). Based on these AUC results our models perform excellently in classifying R-genes (Swets, 1988; Zweig and Campbell, 1993; Semwal et al., 2017; Duhan et al., 2022).

### Precision-recall analysis

**Figure 6:**
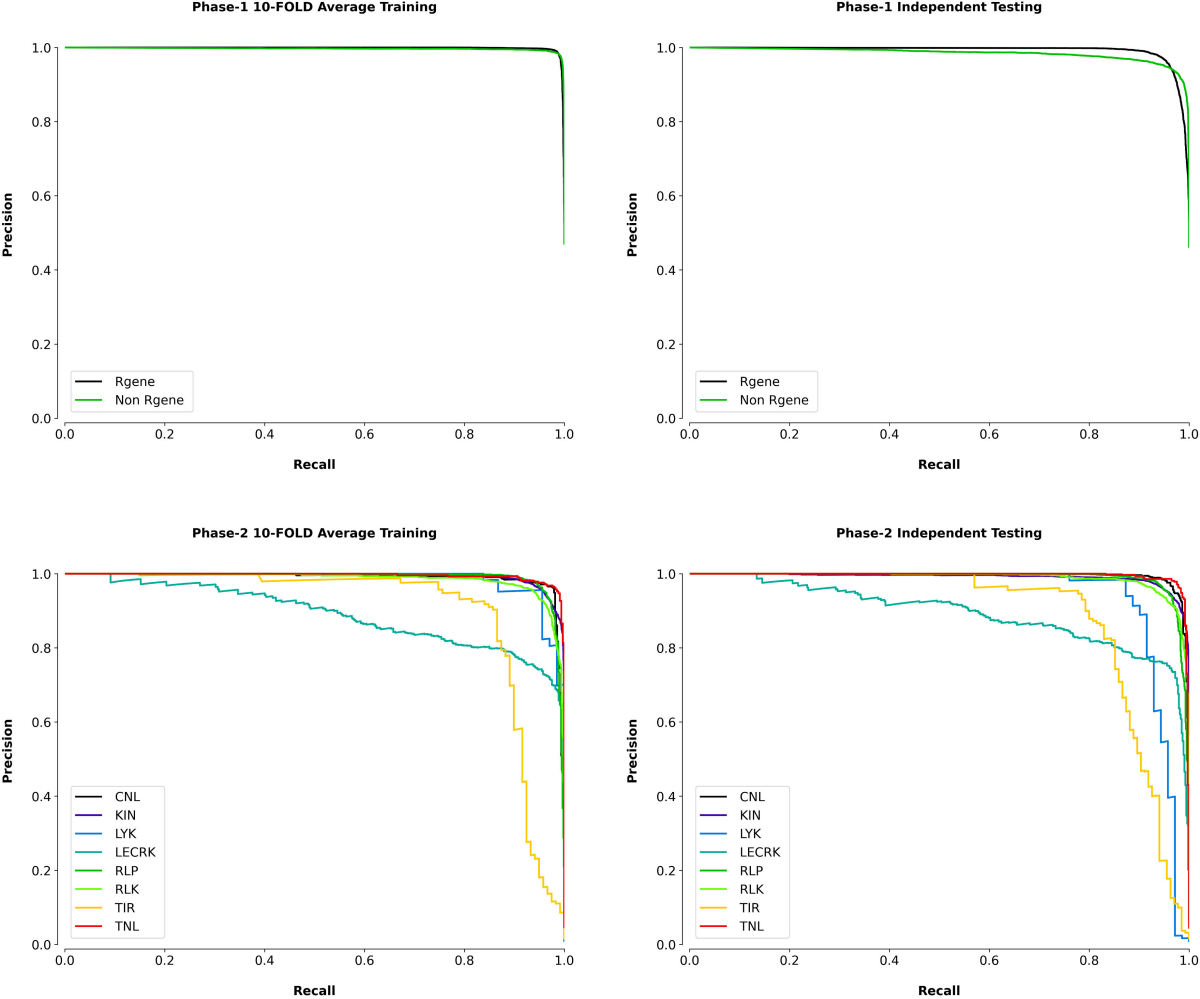
Precision-recall curves for Phase 1 (R-gene identification) and Phase 2 (R-gene classification) of PRGminer, evaluated using 10-fold cross-validation and independent testing datasets. Top Row: Precision-recall curves for Phase 1, which distinguishes between R-genes and non-R-genes. The left plot shows the average 10-fold cross-validation results, while the right plot shows performance on independent testing data. In both cases, high precision and recall values demonstrate that the model is effective in identifying R-genes (black line) and non-R-genes (green line). Bottom Row: Precision-recall curves for Phase 2, where identified R-genes are further classified into subfamilies. The left plot displays the average 10-fold cross-validation results, while the right plot shows the performance on independent testing data. The various colors represent the different R-gene subtypes: CNL (black), KIN (blue), LYK (green), LECRK (yellow), RLP (teal), RLK (purple), TIR (red), and TNL (orange). The curves indicate high precision and recall for most subtypes, although performance varies slightly depending on the R-gene class.

We also plotted the precision-recall curves which is also useful graphical indicator for assessing a classifier’s effectiveness. It is shown on the graph by adjusting the decision criteria and plotting precision on the *y*-axis and recall on the *x*-axis. Precision is the fraction of true positive predictions produced by the classifier out of all positive predictions made by the classifier. The fraction of true positive forecasts among all the real positive cases is measured by recall, also known as sensitivity or true positive rate (TPR). The precision-recall curve provides information about the classifier’s optimistic predictive efficiency since it focuses on the model’s ability to properly predict positive instances while ignoring real negative cases. In our results the precision-recall curve of phase I shows an AUC of 1 square unit in 10-FOLD training and 0.99 on independent dataset. In phase II the average AUC of 8 classes in 10-FOLD training is 0.97 and on independent dataset the average AUC was 0.96. In the precision-recall curve, the optimal classification condition is located at the upper right corner, where the region under the curve covers 1 square unit. In this region, the classifier achieves both precision and recall of 100%. This means the classifier can predict true positives (TP) without any false positives (FP) in its predictions, making it highly reliable. To provide a good classification model, there must be a fair trade-off between accuracy and recall. This trade-off is reflected in the region below the precision-recall curve, which should be as close to 1 square unit as possible. A larger area under the curve indicates better overall performance of the classifier. The precision-recall curve complements the ROC curve, as it provides a different perspective on the classifier’s performance, particularly when dealing with imbalanced datasets where the number of negative cases significantly outweighs the positive cases (Fang et al., 2018; Duhan et al., 2022). The precision-recall curves of the *k*-fold training/testing and on independent data for phase I and phase II are depicted in **Figure 6**.

### Benchmarking with another set of experimental data

**Figure 7:**
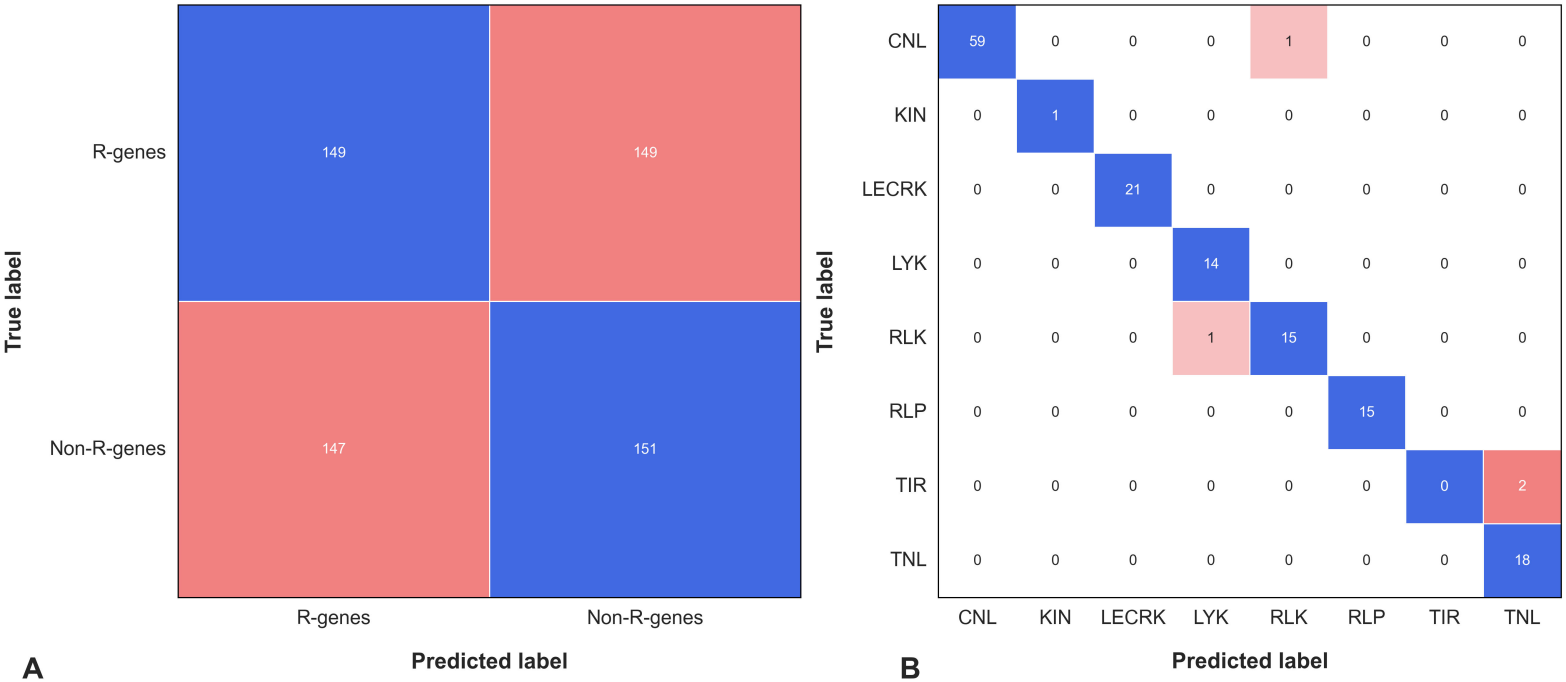
The confusion matrix represents the performance of PRGminer on benchmark dataset containing 149 experimentally validated R-genes and 149 non-R-genes sequences. **(A)** The matrix shows the matrix of Phase I. **(B)** The matrix shows how the predicted labels for the different subfamilies (on the x-axis) compare with the true labels (on the y-axis). The blue color represents the correctly predicted true labels while the tomato color gradient represents the number of sequences incorrectly classified into other subfamilies.

To validate our models further, we obtained 149 experimentally validated resistance genes from the PRGdb database (Calle García et al., 2022) and their corresponding protein sequences from NCBI (https://ncbi.nlm.nih.gov), and also took 149 negative sequences and made a dataset of 298 proteins sequences referred to as Independent Dataset-II. These sequences were then used as input for the PRGminer prediction tool. In the first phase of our validation, out of the 298 sequences predicted 147 as R-genes and 151 as non-R-gene. PRGminer predicted 147 of them correctly as R-genes (resistance genes), while 2 were predicted as non-R-genes and the overall prediction accuracy was high, with over 99.32% accuracy. In the second phase, for the eight different classes, the overall prediction accuracy was still impressively high, with over 96% correct predictions (**Supplementary File 2**). Looking at the class-wise statistics, we found that out of the total 61 sequences in the CNL class, 59 were correctly predicted as CNL. Similarly, all the sequences (1 out of 1) in the KIN class were correctly predicted. For the LECRK class, all 21 sequences were predicted accurately. In the LYK class, 14 out of 14 sequences were correctly predicted, and for the RLK class, 15 out of 17 sequences were accurately predicted. Moreover, all the 15 sequences in the RLP class were correctly identified. However, for the TIR class, none of the 2 sequences were successfully predicted, while for the TNL class, all 18 sequences were accurately predicted. To better visualize the performance of our phase II model, we created a confusion matrix as depicted in **Figure 7**. The comprehensive results for the predictions on this independent dataset-II are presented in **Supplementary File 2**. PRGminer’s strong performance on Independent Dataset-II provides valuable insights into its predictive capabilities. This demonstrates PRGminer’s ability to accurately identify and classify plant resistance genes (R-genes), highlighting its potential as a valuable tool for plant R-genes research. PRGminer accurately predicted experimentally validated R-genes with over 96% accuracy, demonstrating its reliability and effectiveness. It adapts to diverse datasets, highlighting its robustness in classification or R-genes. Its impressive performance across various R-gene classes reflects its ability to discern subtle molecular patterns, underpinning its predictive capacity. Additionally, PRGminer’s capacity to correctly classify R-genes within different classes underscores its versatility and suitability for complex multi-class classification challenges. Although there are areas for improvement, such as the TIR class, the overall performance on Independent Dataset-II accentuates PRGminer’s potential for enhancing R-gene identification and classification.

### Application and use case of PRGminer

**Figure 8:**
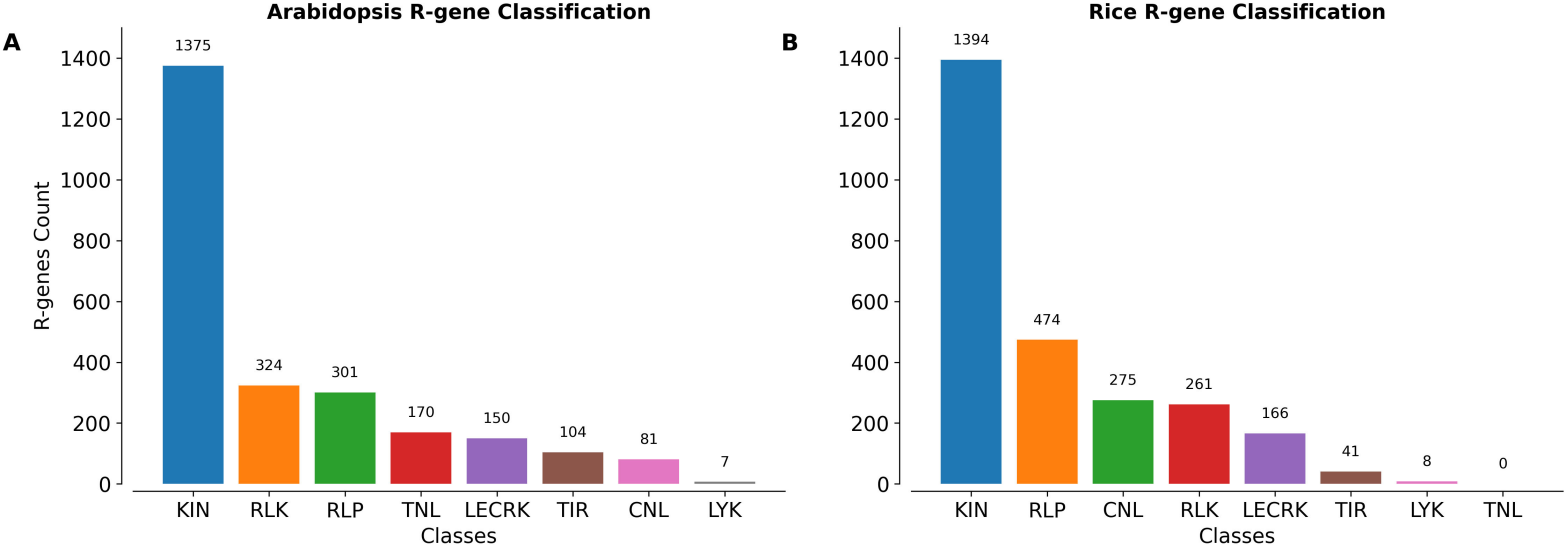
The number of sequences belonging to different classes of R-genes annotated in the **(A)** *Arabidopsis thaliana* and **(B)** *Oryza sativa* Japonica Group proteomes using PRGminer. These R-genes classes include: CNL (Coiled-coil, Nucleotide-binding site, Leucine-rich repeat domains), KIN (Kinase domain), RLP (Leucine-rich repeat and Transmembrane domains with a cytoplasmic region), LECRK (Lectin, Kinase, and Transmembrane domains), RLK (Extracellular Leucine-rich repeat and Kinase domains), LYK (LysM domain, Kinase, and Transmembrane domains), TIR (Toll/interleukin-1 receptor domain), and TNL (Toll/interleukin-1 receptor, Nucleotide-binding site, and Leucine-rich repeat domains).

To demonstrate the application and usability of PRGminer we annotated the *Arabidopsis thalina* and *Oryza sativa* Japonica Group proteomes. We downloaded the recent TAIR 10 and Rice Japonica Group proteomes from Ensembl Plants and analyzed using PRGminer for R-genes prediction and classification. In Arabidopsis: Out of the 48,321 protein sequences, 11,117 duplicate protein sequences were removed using CD-HIT. Finally, 37,204 non-redundant proteins sequences 2,512 sequences were predicted as R-genes while 34,692 as non-R-genes in phase I. Further, in phase -II these R-genes were classified into 8 classes. 1375 R-genes were classified in KIN class followed by 324 in RLK, 301 RLP, 170 TNL, 150 LECRK, 104 TIR, 81 CNL and 7 in LYK respectively. The distribution of R-genes classes in Arabidopsis is depicted in **Figure 8A** and prediction results presented in **Supplementary File 3**. Similarly, in Rice out of 42,582 protein sequences, 4372 duplicate sequences were removed using CD-HIT. Finally, 38,210 non-redundant protein sequences 2,619 sequences were predicted as R-genes while 35,591 sequences were predicted as non-Rgenes in phase-I. Further in phase-II these R-genes were classified into 7 classes, 1394 R-genes were classified in KIN class followed by 474 in RLP, 275 in CNL, 261 in RLK, 166 in LECRK, 41 in TIR and 8 in LYK respectively. The distribution of R-gene classes is depicted in **Figure 8B** and prediction results are presented in **Supplementary File 3**. These results highlighted PRGminer’s ability to correctly predict and classify R-genes in both dicotyledonous and monocotyledonous plants. The tool can be readily applied to other plant species for genome-wide R-gene annotation, facilitating further experimental validation and aiding research in plant disease resistance and functional genomics.

### Comparison of PRGminer with other tools

Several machine learning-based tools, such as NBSPred (Kushwaha et al., 2016) and DRPPP (Pal et al., 2016), were previously used for plant resistance (R) gene prediction. However, these tools are no longer available, so we excluded them from our comparison. Instead, we evaluated PRGminer against PRGdb 4.0’s DRAGO3, an R-gene annotator tool (Calle García et al., 2022). Since DRAGO3 does not provide source code and is only accessible through a web API, we encountered initial difficulties in using it but eventually succeeded.

For benchmarking, we used the Rice proteome containing 38,210 proteins. PRGminer completed the analysis in just 5 minutes, whereas PRGdb required approximately 10–12 minutes. Additionally, PRGminer identified 2,619 R-genes, significantly outperforming PRGdb, which predicted only 1,875 R-genes. A key reason for this difference is PRGdb’s uses domain based precition methods and PRGminer uses a more robust deep learning model.

**Figure 9:**
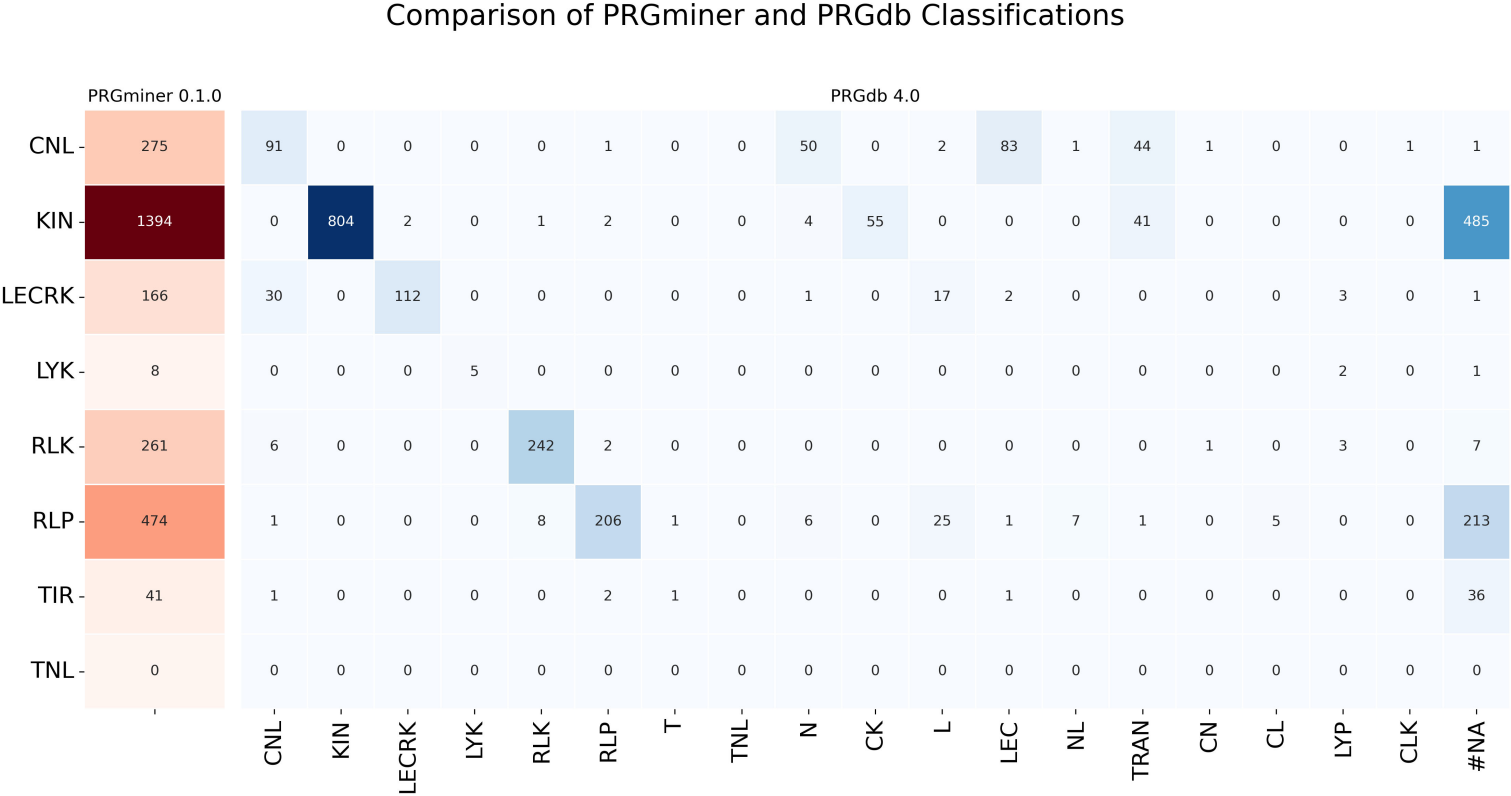
Comparison of PRGminer and PRGdb 4.0 classifications for R-gene annotation in the Rice proteome. The heatmap visualizes the distribution of genes classified by PRGminer (rows) and PRGdb (columns), highlighting differences in classification. PRGminer categorizes genes into eight main classes, while PRGdb assigns some genes to fragmented domain classes. The color gradient represents the count of genes in each category. #NA is the number of genes classified as R-genes by PRGminer but not by PRGdb 4.0.

A detailed class-wise comparison further highlights PRGminer’s advantages. For instance, PRGminer predicted 275 genes in the CNL class, while PRGdb identified only 91. The additional genes predicted by PRGdb were scattered into fragmented categories such as 50 in “N,” 83 in “NL,” 44 in “CN,” 2 in “L,” and a few in CL and NLK, rather than being classified under a broader. Similarly, PRGminer predicted 1,394 KIN genes, whereas PRGdb assigned only 804 to KIN while classifying the remaining genes across various minor subclasses. Other R-gene classes such as RLK, RLP, and LECRK followed a similar trend, with PRGminer consistently demonstrating a more comprehensive and streamlined classification. These results indicate that PRGminer is not only faster but also provides a more precise annotation of R-genes. The complete distribution of predicted genes across different classes is depicted in **Figure 9**.

## Conclusion

In this study, we developed PRGminer for the prediction of plant resistance proteins using convolution neural networks. PRGminer was tested on a dataset of experimentally validated plant resistance genes, and it accurately predicted over 96% of the genes. This shows that PRGminer is a reliable and precise tool for predicting R-genes. PRGminer has the potential to be a valuable tool for the research community, particularly in the domain of crop protection. By accurately predicting plant resistance proteins, PRGminer can help scientists to identify and develop new crop varieties that are resistant to pests and diseases. In addition to its potential for crop protection, PRGminer could also be used to advance our understanding of the intricate mechanisms governing plant defense systems. By identifying the key features of resistance proteins, PRGminer could help scientists to develop new strategies for engineering crops with enhanced disease resistance.

In the future, as more diverse and comprehensive datasets become available, we plan to augment the tool’s Phase II by incorporating additional R-gene classes. This evolutionary step will further refine PRGminer’s predictive capacity, enabling it to tackle a broader spectrum of resistance genes with enhanced precision. Which will allow PRGminer to predict a wider range of plant resistance proteins with even greater accuracy. PRGminer’s development has the potential to advance crop protection and our understanding of plant defense systems.

## Data Availability

The trained models are provided both as a web server and as a standalone tool. The web server is accessible at https://kaabil.net/prgminer, and the standalone tool can be downloaded from https://github.com/usubioinfo/PRGminer.

## Supplementary material

Supplementary File 1 Results for 10-fold cross validation statistics of all the sequence representations tried in this study.

Supplementary File 2 Results for benchmarking of the PRGminer on experimentally proven R-genes; independent dataset-II.

Supplementary File 3
*Arabidopsis thaliana* and *Oryza sativa* Japonica group proteome annotation using PRGminer.

## References

[B1] Abadi M. Barham P. Chen J. Chen Z. Davis A. Dean J.. (2016). “Tensorflow: A system for large-scale machine learning,” in 12th USENIX symposium on operating systems design and implementation (OSDI 16). 265–283. Available online at: https://www.usenix.org/system/files/conference/osdi16/osdi16-abadi.pdf.

[B2] Abma B. J. M. (2009). Evaluation of requirements management tools with support for traceability-based change impact analysis. Available online at: https://www.usenix.org/system/files/conference/osdi16/osdi16-abadi.pdf.

[B3] Altschul S. F. Gish W. Miller W. Myers E. W. Lipman D. J. (1990). Basic local alignment search tool. J. Mol. Biol. 215, 403–410. doi: 10.1016/s0022-2836(05)80360-2 2231712

[B4] Andolfo G. D’agostino N. Frusciante L. Ercolano M. R. (2021a). The tomato interspecific NB-LRR gene arsenal and its impact on breeding strategies. Genes 12, 184. doi: 10.3390/GENES12020184 33514027 PMC7911644

[B5] Andolfo G. Di Donato A. Chiaiese P. De Natale A. Pollio A. Jones J. D. G.. (2019). Alien domains shaped the modular structure of plant NLR proteins. Genome Biol. Evol. 11, 3466–3477. doi: 10.1093/GBE/EVZ248 31730154 PMC7145615

[B6] Andolfo G. Dohm J. C. Himmelbauer H. (2022). Prediction of NB-LRR resistance genes based on full-length sequence homology. Plant J. 110, 1592–1602. doi: 10.1111/TPJ.15756 35365907 PMC9322396

[B7] Andolfo G. Ercolano M. R. (2015). Plant innate immunity multicomponent model. Front. Plant Sci. 6. doi: 10.3389/FPLS.2015.00987/BIBTEX PMC4643146 26617626

[B8] Andolfo G. Jupe F. Witek K. Etherington G. J. Ercolano M. R. Jones J. D. G. (2014). Defining the full tomato NB-LRR resistance gene repertoire using genomic and cDNA RenSeq. BMC Plant Biol. 14, 120. doi: 10.1186/1471-2229-14-120 24885638 PMC4036795

[B9] Andolfo G. Sánchez C. S. Cañizares J. Pico M. B. Ercolano M. R. (2021b). Large-scale gene gains and losses molded the NLR defense arsenal during the Cucurbita evolution. Planta 254, 1–14. doi: 10.1007/S00425-021-03717-X/TABLES/4 34559316 PMC8463517

[B10] Andolfo G. Villano C. Errico A. Frusciante L. Carputo D. Aversano R.. (2020). Inferring RPW8-NLRs’s evolution patterns in seed plants: case study in Vitis vinifera. Planta 251, 1–13. doi: 10.1007/S00425-019-03324-X/FIGURES/6 31823009

[B11] Barchi L. Pietrella M. Venturini L. Minio A. Toppino L. Acquadro A.. (2019). A chromosome-anchored eggplant genome sequence reveals key events in Solanaceae evolution. Sci. Rep. 9, 11769. doi: 10.1038/S41598-019-47985-W 31409808 PMC6692341

[B12] Boughorbel S. Jarray F. El-Anbari M. (2017). Optimal classifier for imbalanced data using Matthews Correlation Coefficient metric. PloS One 12, e0177678. doi: 10.1371/journal.pone.0177678 28574989 PMC5456046

[B13] Calle García J. Guadagno A. Paytuvi-Gallart A. Saera-Vila A. Amoroso C. G. D’esposito D.. (2022). PRGdb 4.0: an updated database dedicated to genes involved in plant disease resistance process. Nucleic Acids Res. 50, D1483–D1490. doi: 10.1093/NAR/GKAB1087 34850118 PMC8729912

[B14] Camacho C. Coulouris G. Avagyan V. Ma N. Papadopoulos J. Bealer K.. (2009). BLAST+: architecture and applications. BMC Bioinf. 10, 1–9. doi: 10.1186/1471-2105-10-421/FIGURES/4 PMC2803857 20003500

[B15] Capistrano-Gossmann G. G. Ries D. Holtgräwe D. Minoche A. Kraft T. Frerichmann S. L. M.. (2017). Crop wild relative populations of Beta vulgaris allow direct mapping of agronomically important genes. Nat. Commun. 8, 15708. doi: 10.1038/NCOMMS15708 28585529 PMC5467160

[B16] Di Donato A. Andolfo G. Ferrarini A. Delledonne M. Ercolano M. R. Crease T. (2017). Investigation of orthologous pathogen recognition gene-rich regions in solanaceous species. Genome 60, 850–859. doi: 10.1139/GEN-2016-0217 28742982

[B17] Duhan N. Norton J. M. Kaundal R. (2022). deepNEC: a novel alignment-free tool for the identification and classification of nitrogen biochemical network-related enzymes using deep learning. Brief Bioinform. 23, bbac071. doi: 10.1093/bib/bbac071 35325031

[B18] Eddy S. R. (2011). Accelerated profile HMM searches. PloS Comput. Biol. 7, e1002195. doi: 10.1371/JOURNAL.PCBI.1002195 22039361 PMC3197634

[B19] Fang M. Lei X. Guo L. (2018). A survey on computational methods for essential proteins and genes prediction. Curr. Bioinform. 14, 211–225. doi: 10.2174/1574893613666181112150422

[B20] FAO publications catalogue 2023 (2023). doi: 10.4060/CC7285EN

[B21] Finn R. D. Bateman A. Clements J. Coggill P. Eberhardt R. Y. Eddy S. R.. (2014). Pfam: the protein families database. Nucleic Acids Res. 42, D222–30. doi: 10.1093/NAR/GKT1223 24288371 PMC3965110

[B22] Fu L. Niu B. Zhu Z. Wu S. Li W. (2012). CD-HIT: accelerated for clustering the next-generation sequencing data. Bioinformatics 28, 3150–3152. doi: 10.1093/BIOINFORMATICS/BTS565 23060610 PMC3516142

[B23] Goodstein D. M. Shu S. Howson R. Neupane R. Hayes R. D. Fazo J.. (2012). Phytozome: A comparative platform for green plant genomics. Nucleic Acids Res. 40, D1178–D1186. doi: 10.1093/NAR/GKR944 22110026 PMC3245001

[B24] Han G. Z. (2019). Origin and evolution of the plant immune system. New Phytol. 222, 70–83. doi: 10.1111/NPH.15596 30575972

[B25] Jones J. D. G. Dangl J. L. (2006). The plant immune system. Nature 444, 323–329. doi: 10.1038/NATURE05286 17108957

[B26] Jupe F. Witek K. Verweij W. Śliwka J. Pritchard L. Etherington G. J.. (2013). Resistance gene enrichment sequencing (RenSeq) enables reannotation of the NB-LRR gene family from sequenced plant genomes and rapid mapping of resistance loci in segregating populations. Plant J. 76, 530–544. doi: 10.1111/TPJ.12307 23937694 PMC3935411

[B27] Käll L. Krogh A. Sonnhammer E. L. L. (2004). A combined transmembrane topology and signal peptide prediction method. J. Mol. Biol. 338, 1027–1036. doi: 10.1016/j.jmb.2004.03.016 15111065

[B28] Kaundal R. Loaiza C. D. Duhan N. Flann N. (2022). deepHPI: a comprehensive deep learning platform for accurate prediction and visualization of host–pathogen protein–protein interactions. Brief Bioinform. 23, bbac125. doi: 10.1093/bib/bbac125 35511057

[B29] Kinsella R. J. Kähäri A. Haider S. Zamora J. Proctor G. Spudich G.. (2011). Ensembl BioMarts: a hub for data retrieval across taxonomic space. Database 2011, bar030. doi: 10.1093/DATABASE/BAR030 21785142 PMC3170168

[B30] Krogh A. Larsson B. Von Heijne G. Sonnhammer E. L. L. (2001). Predicting transmembrane protein topology with a hidden Markov model: application to complete genomes. J. Mol. Biol. 305, 567–580. doi: 10.1006/JMBI.2000.4315 11152613

[B31] Kushwaha S. K. Åhman I. Bengtsson T. (2021). ResCap: plant resistance gene prediction and probe generation pipeline for resistance gene sequence capture. Bioinf. Adv. 1, vbab033. doi: 10.1093/BIOADV/VBAB033 PMC9710708 36700100

[B32] Kushwaha S. K. Chauhan P. Hedlund K. Ahren D. (2016). NBSPred: a support vector machine-based high-throughput pipeline for plant resistance protein NBSLRR prediction. Bioinformatics 32, 1223–1225. doi: 10.1093/BIOINFORMATICS/BTV714 26656003

[B33] Li W. Godzik A. (2006). Cd-hit: A fast program for clustering and comparing large sets of protein or nucleotide sequences. Bioinformatics 22, 1658–1659. doi: 10.1093/bioinformatics/btl158 16731699

[B34] Li P. Quan X. Jia G. Xiao J. Cloutier S. You F. M. (2016). RGAugury: A pipeline for genome-wide prediction of resistance gene analogs (RGAs) in plants. BMC Genomics 17, 1–10. doi: 10.1186/S12864-016-3197-X/FIGURES/4 27806688 PMC5093994

[B35] Lupas A. Dyke M.V. Stock J. (1991). Predicting coiled coils from protein sequences. New Ser. 252, 1162–1164. doi: 10.1126/science.252.5009.1162 2031185

[B36] Marone D. Russo M. A. Laidò G. De Leonardis A. M. Mastrangelo A. M. (2013). Plant nucleotide binding site-leucine-rich repeat (NBS-LRR) genes: active guardians in host defense responses. Int. J. Mol. Sci. 14, 7302–7326. doi: 10.3390/IJMS14047302 23549266 PMC3645687

[B37] Nguyen Q. M. Iswanto A. B. B. Son G. H. Kim S. H. (2021). Recent advances in effector-triggered immunity in plants: new pieces in the puzzle create a different paradigm. Int. J. Mol. Sci. 22, 4709. doi: 10.3390/IJMS22094709 33946790 PMC8124997

[B38] Pal T. Jaiswal V. Chauhan R. S. (2016). DRPPP: A machine learning based tool for prediction of disease resistance proteins in plants. Comput. Biol. Med. 78, 42–48. doi: 10.1016/J.COMPBIOMED.2016.09.008 27658260

[B39] Pedregosa F. Michel V. Grisel Oliviergrisel O. Blondel M. Prettenhofer P. Weiss R.. (2011). Scikit-learn: machine learning in python. Available online at: http://scikit-learn.sourceforge.net (Accessed February 2, 2021).

[B40] Peng Y. Van Wersch R. Zhang Y. (2018). Convergent and divergent signaling in PAMP-triggered immunity and effector-triggered immunity. Mol. Plant-Microbe Interact. 31, 403–409. doi: 10.1094/MPMI-06-17-0145-CR/ASSET/IMAGES/LARGE/MPMI-06-17-0145-CR_F1.JPEG 29135338

[B41] Petersen T. N. Brunak S. Von Heijne G. Nielsen H. (2011). SignalP 4.0: discriminating signal peptides from transmembrane regions. Nat. Methods 8, 785–786. doi: 10.1038/NMETH.1701 21959131

[B42] Ray S. K. Macoy D. M. Kim W. Y. Lee S. Y. Kim M. G. (2019). Role of RIN4 in regulating PAMP-triggered immunity and effector-triggered immunity: current status and future perspectives. Mol. Cells 42, 503–511. doi: 10.14348/MOLCELLS.2019.2433 31362467 PMC6681865

[B43] Restrepo-Montoya D. Brueggeman R. McClean P. E. Osorno J. M. (2020). Computational identification of receptor-like kinases “rLK” and receptor-like proteins “rLP” in legumes. BMC Genomics 21, 1–17. doi: 10.1186/S12864-020-06844-Z/TABLES/9 PMC7333395 32620079

[B44] Ristaino J. B. Anderson P. K. Bebber D. P. Brauman K. A. Cunniffe N. J. Fedoroff N. V.. (2021). The persistent threat of emerging plant disease pandemics to global food security. Proc. Natl. Acad. Sci. U S A 118, e2022239118. doi: 10.1073/PNAS.2022239118/-/DCSUPPLEMENTAL 34021073 PMC8201941

[B45] Semwal R. Aier I. Raj U. Varadwaj P. K. (2017). Pharmadoop: a tool for pharmacophore searching using Hadoop framework. Network Modeling Anal. Health Inf. Bioinf. 6, 1–9. doi: 10.1007/s13721-017-0161-x

[B46] Skendžić S. Zovko M. Živković I. P. Lešić V. Lemić D. (2021). The impact of climate change on agricultural insect pests. Insects 12, 440. doi: 10.3390/INSECTS12050440 34066138 PMC8150874

[B47] Stehman S. V. (1997). Selecting and interpreting measures of thematic classification accuracy. Remote Sens Environ. 62, 77–89. doi: 10.1016/S0034-4257(97)00083-7

[B48] Steuernagel B. Jupe F. Witek K. Jones J. D. G. Wulff B. B. H. (2015). NLR-parser: rapid annotation of plant NLR complements. Bioinformatics 31, 1665–1667. doi: 10.1093/BIOINFORMATICS/BTV005 25586514 PMC4426836

[B49] Sun Y. Qiao Z. Muchero W. Chen J. G. (2020a). Lectin receptor-like kinases: the sensor and mediator at the plant cell surface. Front. Plant Sci. 11. doi: 10.3389/FPLS.2020.596301/BIBTEX PMC7758398 33362827

[B50] Sun Y. Zhu Y. X. Balint-Kurti P. J. Wang G. F. (2020b). Fine-tuning immunity: players and regulators for plant NLRs. Trends Plant Sci. 25, 695–713. doi: 10.1016/J.TPLANTS.2020.02.008 32526174

[B51] Swets J. A. (1988). Measuring the accuracy of diagnostic systems. Science 240, 1285–1293. doi: 10.1126/science.3287615 3287615

[B52] Tang D. Wang G. Zhou J. M. (2017). Receptor kinases in plant-pathogen interactions: more than pattern recognition. Plant Cell 29, 618. doi: 10.1105/TPC.16.00891 28302675 PMC5435430

[B53] Tørresen O. K. Star B. Mier P. Andrade-Navarro M. A. Bateman A. Jarnot P.. (2019). Tandem repeats lead to sequence assembly errors and impose multi-level challenges for genome and protein databases. Nucleic Acids Res. 47, 10994–11006. doi: 10.1093/NAR/GKZ841 31584084 PMC6868369

[B54] Valverde-Albacete F. J. Carrillo-de-Albornoz J. Peláez-Moreno C. (2013). “A proposal for new evaluation metrics and result visualization technique for sentiment analysis tasks,” in Lecture Notes in Computer Science (including subseries Lecture Notes in Artificial Intelligence and Lecture Notes in Bioinformatics) (Springer, Berlin, Heidelberg), 41–52. doi: 10.1007/978-3-642-40802-1_5

[B55] Van Ooijen G. Mayr G. Kasiem M. M. A. Albrecht M. Cornelissen B. J. C. Takken F. L. W. (2008). Structure-function analysis of the NB-ARC domain of plant disease resistance proteins. J. Exp. Bot. 59, 1383–1397. doi: 10.1093/JXB/ERN045 18390848

[B56] Wang Y. Bouwmeester K. (2017). L-type lectin receptor kinases: New forces in plant immunity. PloS Pathog 13, e1006433. doi: 10.1371/JOURNAL.PPAT.1006433 28817713 PMC5560540

[B57] Wang Y. Wang P. Guo Y. Huang S. Chen Y. Xu L. (2021). prPred: A predictor to identify plant resistance proteins by incorporating k-spaced amino acid (Group) pairs. Front. Bioeng Biotechnol. 8. doi: 10.3389/FBIOE.2020.645520/BIBTEX PMC7859348 33553134

[B58] Yates A. D. Allen J. Amode R. M. Azov A. G. Barba M. Becerra A.. (2022). Ensembl Genomes 2022: an expanding genome resource for non-vertebrates. Nucleic Acids Res. 50, D996–D1003. doi: 10.1093/NAR/GKAB1007 34791415 PMC8728113

[B59] Zdobnov E. M. Apweiler R. (2001). InterProScan–an integration platform for the signature-recognition methods in InterPro. Bioinformatics 17, 847–848. doi: 10.1093/BIOINFORMATICS/17.9.847 11590104

[B60] Zhou J. M. Yang W. C. (2016). Receptor-like kinases take center stage in plant biology. Sci. China Life Sci. 59, 863–866. doi: 10.1007/S11427-016-5112-8 27604522

[B61] Zweig M. H. Campbell G. (1993). Receiver-operating characteristic (ROC) plots: a fundamental evaluation tool in clinical medicine. Clin. Chem. 39, 561–577. doi: 10.1093/clinchem/39.4.561 8472349

